# Moderate Effects of the Arginine to Histidine R47H Variant of the Triggering Receptor Expressed on Myeloid Cells 2 (TREM2) on Bone Structure in Male and Female Mice: Insights from the Four Core Genotypes mice

**DOI:** 10.21203/rs.3.rs-9076483/v1

**Published:** 2026-03-18

**Authors:** Gabriel Ramirez, Dayanara Hernandez, Alix Teal, Lakshmi Chellaganapathy, Roquelina Pianeta, Dyann M Segvich, Joseph M. Wallace, Lilian I. Plotkin

**Affiliations:** Indiana University School of Medicine; Indiana University School of Medicine; Indiana University School of Medicine; Indiana University School of Medicine; Indiana University School of Medicine; Purdue University; Purdue University; Indiana University School of Medicine

## Abstract

**Background:**

The Triggering Receptor Expressed on Myeloid Cells 2 (TREM2) gene is expressed in cells of the hematopoietic lineage, like microglia and osteoclasts. A TREM2 gene variant known as TREM2-R47H is associated with an increased risk of developing Alzheimer’s disease (AD). Previous studies have shown sex-dimorphic bone and muscle consequences that are associated with the TREM2 variant. Sex chromosomes have also been shown to play a key contributor to skeletal mass and bone strength. Due to the sex-dimorphic bone and skeletal muscle phenotype exhibited by mice expressing the TREM2 gene variant, we investigated the role of chromosomal (XX vs XY) or gonadal (ovaries vs testes) sex.

**Methods:**

Four Core Genotypes (FCG) C57Bl/6J mice expressing the TREM2-R47H variant were mated to obtain TREM2 wildtype (TREM2^+/+^, WT) and TREM2^R47H/+^ FCG mice. Four to 5.5-month-old gonadal male (XXT and XYT) and female (XXO and XYT) mice were analyzed. Body weight and bone mineral density were initially measured at baseline and endpoint (5.5 months of age) by DXA/Piximus. Micro-computed tomography, dynamic histomorphometry, 3-point bending test (mechanical properties), and bone turnover markers were measured at the endpoint. Two-way ANOVA analyses were performed through Prism 10 to identify the contributions of chromosome sex, the presence of the TREM2-R47H variant, and their interaction, separately for each gonadal sex.

**Results:**

Gonadal males: chromosome sex (XX/XY) effects are found for several bone structural parameters in femur and lumbar vertebra 5, whereas there was an interaction between gonadal sex and chromosome sex for other structural measurements in both bones by μCT. Overall, values are higher for TREM2^R47H/+^ than WT for XYT, but not XXT mice, suggesting that the TREM2 genotype effects depend on the presence of the Y chromosome. Mechanical testing shows chromosome sex effects, with higher overall values for XXT mice. Bone formation on the femur cortex and serum formation/resorption markers were unchanged, suggesting that structural changes result from bone modeling/remodeling at an earlier age. Gonadal females: Chromosome sex affects body weight gain (higher in XYO than XXO mice), but no bone mineral density accrual. Chromosome sex affects total lean mass (XYO > XXO) with chromosome sex x TREM2 genotype interaction and differences in total/%fat mass (TREM2^R47H/+^<WT), and %lean mass (TREM2^R47H/+^>WT) only for XYO mice. Chromosome sex affects distal femur volumetric bone mass (XYO > XXO), but the TREM2 genotype influences lumbar vertebra trabecular number and separation, which trended higher in TREM2^R47H/+^ vs WT mice for sex complement. Chromosome sex influences femur cortical bone, with overall higher values in XXO mice, independent of TREM2 genotype. Mechanical testing parameters also were XXO > XYO mice. Femur cortical bone formation is higher on the endocortical but lower on the periosteal surface in XXO vs XYO (chromosome sex effect). The opposite effects on the bone surfaces might explain the unchanged serum bone formation marker, Procollagen Type 1 N-terminal propeptide (P1NP). Yet, chromosome sex affects the levels of the resorption marker, C-terminal telopeptide of type 1 collagen (CTX-1), which were lower in XXO mice.

**Conclusion:**

Our findings suggest that chromosome sex partially affects the consequences of expression of the TREM2-R47H variant on bone structure, whereas the outcomes of the gene variant depend on the mouse gonadal sex.

## Plain Language summary

Alzheimer’s disease (AD) is the most common form of dementia and currently affects 6.9 million Americans. AD has also been associated with affecting women at a higher rate compared to men, and recent studies have suggested the Trigger Receptor Expressed on Myeloid Cells 2 (TREM2) R47H point mutation gene may play a key role in AD and in bone integrity. To uncover the role of the TREM2 variant, a transgenic mouse model was used to tease out the effects of sex chromosomes on the consequences of the presence of the TREM2-R47H gene within each gonadal sex.

## Introduction

The most common form of dementia is Alzheimer’s disease (AD), as it accounts for 60 to 80% dementia cases [1]. AD is characterized by memory decline and decreased executive function. Pathologically, patients with AD display amyloid-*β* (A*β*) plaques and tau tangles in the brain. AD manifests in many forms, such as early-onset and late-onset Alzheimer’s disease, EOAD and LOAD, respectively. LOAD is characterized developing AD symptoms at 65 years of age, and accounts for at least 90% of Alzheimer’s disease cases. Variants of eleven different genes have been associated with the development of AD. One of them is the Trigger Receptor Expressed on Myeloid Cells 2 (TREM2) [2–4]. Among these variants, the arginine to histidine replacement in position 47 (R47H) has been associated with both cognitive and skeletal deficits. Further, the TREM2-R47H variant has been linked to age- and sex-dependent effects in altering bone and skeletal muscle mass and strength in mice [5].

As the lifespan increases in humans, an emphasis has been placed on understanding the effects of LOAD and the comorbidities associated with AD [6]. Recent studies have shown a connection between AD and musculoskeletal diseases - osteoporosis and osteo- and rheumatoid arthritis [7–10]. Based on this, AD is considered a global disease, as clinical studies have shown a significant correlation between cognitive decline, bone loss, and fracture risk in women [11]. Another study showed a distinct sex difference, where women with low femoral neck BMD were associated with 2 times the risk of AD compared to men with low femoral neck BMD [12]. These clinical findings express the importance of understanding the correlation between AD and bone tissue loss, and how chromosomal sex and sex hormones can affect the pathology of the disease.

In an effort to understand the basis for the sex differences in the TREM2^R47H/+^ model, adult, 4-month-old mice were gonadectomized, and the musculoskeletal phenotype was studied 5 weeks later [13]. These studies also showed a sexual dimorphic response to sex steroid removal, with an ameliorated effect of ovariectomy in TREM2^R47H/+^ females compared to wild type littermates, and minimal effects of the genotype on the consequences of orchiectomy. Further, these studies showed that the presence of different sex steroids does not completely explain the difference between males and females expressing the TREM2 variant.

We recently found that sex chromosomes contribute as independent factors to musculoskeletal mass and bone strength in the Four-Core Genotypes (FCG) murine model [14]. This evidence prompted the leading question of how the TREM2-R47H variant is affected by chromosomal (XY vs XX) sex in each gonadal (testes or ovaries) sex. Through breeding XY mice with testes (XYT) FCG male with TREM2^R47H/+^ female (XXO) mice, we generated 8 unique genotypes expressing wild type or the TREM2-R47H variant together with the four gonadal/chromosomal sex combinations.

By measuring body weight and composition, and bone architecture, integrity and density, we sought to determine whether chromosomal sex is a prominent variable in the sex differences seen in AD associated with TREM2 within the FCGxTREM2^R47H/+^ mouse line. Our results suggest that chromosomal sex has a limited effect in the context of AD associated with the TREM2 variant, with gonadal females only experiencing a TREM2-R47H effect in the vertebral microarchitecture. On the other hand, interactions between chromosomal sex and the TREM2 gene affected gonadal male femur and vertebral microarchitecture and area, while chromosomal sex and TREM2 gene interactions affected body composition in gonadal females. Our evidence emphasizes the contribution of chromosome sex to the musculoskeletal phenotype of mice with testes or ovaries, with limited effects on the consequences of expression of the TREM2-R47H variant.

## Materials and Methods

### Animal generation

**Figure 1:**
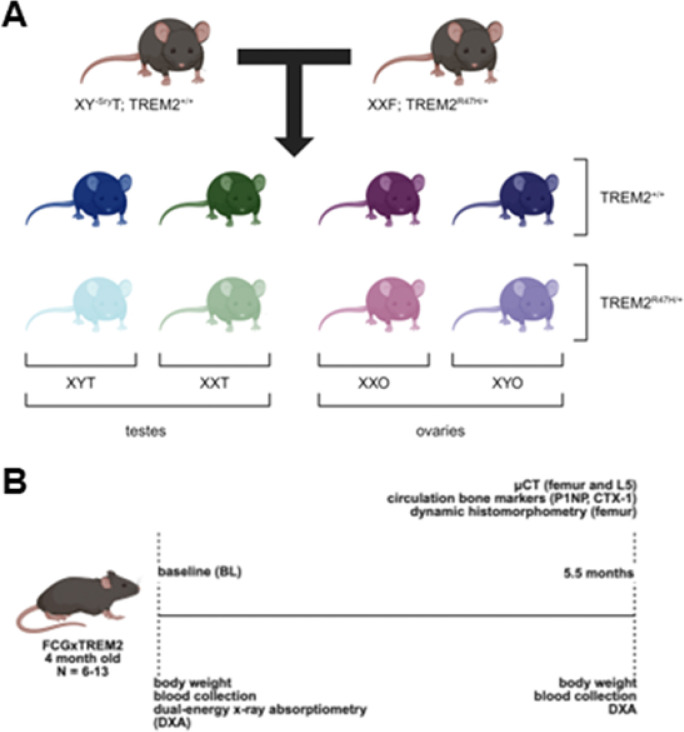
Schematic representation of the generation of the FCGxTREM2 mice and phenotypic characterization of the animals. **(A)** Mice were generated by mating XYT gonadal male TREM2^+/+^ mouse, resulting in animals of the eight genotypes. **(B)** Schematic representation of the timeline for the reported study. Body weight, composition and bone parameters were analyzed at the indicated ages.

B6.Cg-Tg(Sry)2Ei Srydl1Rlb/ArnoJ [15] mice lacking the Sry gene (XY^− *Sry*^) and overexpressing it in chromosome 3 (XY mice with testes or XYT) were mated with female C57BL/6J mice expressing one allele of the TREM2-R47H variant (TREM2^R47H/+^ mice) to generate the wild type and TREM2^R47H/+^ of the four core genotypes (Fig. 1A).

The gonadal sex of the mice was identified at weaning by the external sexual characteristics and confirmed by PCR [16]. The presence of the wild type allele and the TREM2-R47H variant was determined by PCR, as published [17]. All animals were maintained under standardized conditions (5 mice/cage, diet and water *ad libitum*; 12-hour light/dark cycle). Mice (up to 5/cage) were fed with Teklad Global 18% protein extruded rodent diet (20018SX) and water *ad libitum*. All procedures were approved by the Institutional Animal Care and Use Committee of Indiana University School of Medicine. All investigators were blinded to the mouse genotype at the time the analyses were performed.

### Bone mineral density (BMD)

Total (whole body minus head and tail), femur (entire femur), and spine (L1-L6 lumbar vertebrae) BMD was assessed by Dxa/Piximus at 4 months and 5.5 months (Fig. 1B) [18]. Calibration was performed using a standard control phantom, as recommended by the manufacturer.

### Microcomputed tomography (μCT)

L5 vertebrae were scanned in a Scanco μCT-35 system (Scanco Medical AG, Brüttisellen, Switzerland) using the following parameters: 10μm voxel size, 55kV, 120 mA, and exposure time of 151ms, as previously published [16].

Left femurs were scanned using a Bruker Skyscan 1272 at isotropic 10 μm voxel size (70 kV, 142 μA, 0.7-degree angle step, 2 frames averaged), as published [16]. Trabecular regions of interest (ROIs) were chosen as 1 mm extending proximally from the proximal edge of the distal growth plate, and cancellous architecture in each ROI was quantified using custom processing functions in CT Analyzer (CTAn). Cortical properties were measured within a 0.1 mm cortical ROI taken from the central midshaft (approximately 50% of femoral length) and analyzed with a custom MATLAB program (MathWorks, Inc. Natick, MA). After scans, bones were re-wrapped in PBS-soaked gauze and stored at −20°C until bending tests were performed.

### Mechanical testing

The biomechanical properties for the femoral cortical bone were assessed in hydrated bones using a 3-point bending test in displacement control at a rate of 0.025 mm/sec using an ElectroForce 5500 loading device (TA Instruments, New Castle, DE, United States), as reported [19]. Force and displacement were recorded during each test and, using μCT data to determine the moment of inertia about the bending axis (IML) and the furthest distance from the centroid of the bone to the tensile surface, were used to calculate estimated material-level properties. Yield point was defined using the 0.2% offset method on the stress-strain curve and then mapped back to the force-displacement curve.

### Dynamic Histomorphometry

Mice were injected with calcein (20mg/kg; Sigma) and alizarin red (20mg/kg; Sigma) intraperitoneally 7 and 2 days, respectively, prior to euthanasia. The right femora were fixed in 10% neutral buffered formalin and embedded in methyl methacrylate, as reported [20]. Dynamic histomorphometry was performed on femur mid-diaphysison the periosteal and endocortical surfaces and on trabecular bone of the distal femur. Histomorphometric analysis was performed using OsteoMeasure high resolution digital video system (OsteoMetrics Inc., Decatur, GA, USA), and reported following the ASBMR Histomorphometry Nomenclature Committee report [21].

### Statistical analyses

Data were analyzed by two-way ANOVA separately for each gonadal sex, using GraphPad, with chromosomal sex and TREM2 genotype as the two independent variables. Tukey’s multiple comparisons test was used as post-hoc tests. P values < 0.05 are considered significant, and values between 0.073 and 0.05 are considered indicative of tendencies towards significant. P values for the overall two-way ANOVA results are displayed in **Supplementary Table 1–2**, and p values between 0.073 and 0.05, resulting from the post-hoc analyses, are indicated in each graph.

## Results

### Chromosome sex affects body weight and composition and bone mineral density in 4- and 5.5-month-old mice.

**Figure 2:**
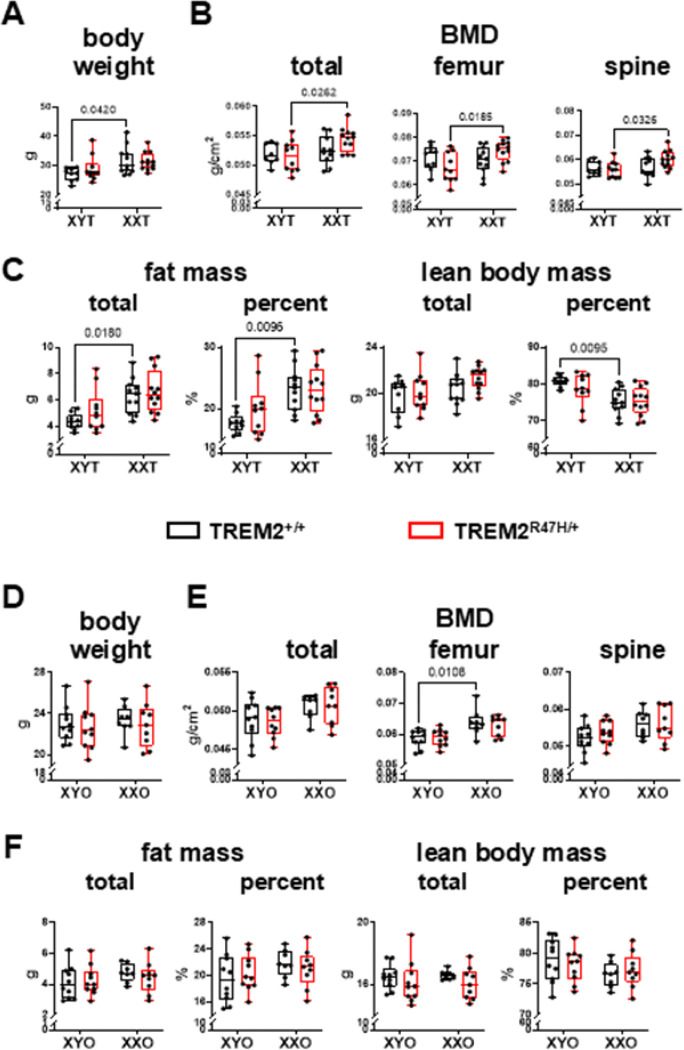
TREM2-R47H variant and chromosome sex effects on body weight/composition andBMD in 4-month-old mice Body weight **(A,D)**, bone mineral density (BMD) **(B,E)** and body composition **(C,F)** were measured in live animals by Dxa/Piximus at 4 months of age in gonadal male **(A-C)** and female **(D-F)** mice. Box boundaries indicate 25^th^ to 75^th^ percentile, the horizontal lines correspond to the median, vertical lines indicate minimum to maximum, and each dot corresponds to an individual sample. p values for the two-way ANOVA analyses, with chromosomal sex and TREM2 genotype as the 2 variables, is shown in **Supplementary Table 1**. Lines indicate pairwise comparisons (Tukey’s multiple comparison test) with in values p < 0.073. N=8–11/group.

**Figure 3:**
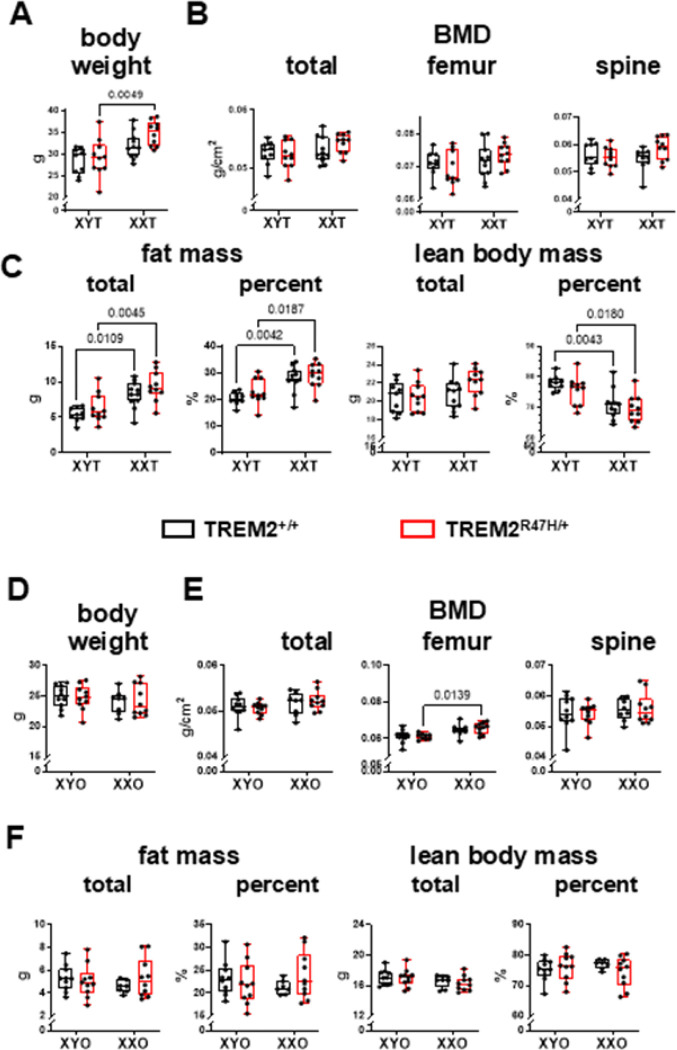
Body weight/composition and BMD are differentialy affected by the TREM2 genotype in 5.5-month-old mice. Body weight (**A,D**), bone mineral density (BMD) (**B,E**) and body composition (**C,F**) were measured in live animals by Dxa/Piximus at 5.5 months of age in gonadal male (**A-C**) and female (**D-F**) mice. Box boundaries indicate 25^th^ to 75^th^ percentile, the horizontal lines correspond to the median, vertical lines indicate minimum to maximum, and each dot corresponds to an individual sample. p values for the two-way ANOVA analyses, with chromosomal sex and TREM2 genotype as the 2 variables, is shown in **Supplementary Table 1**. Lines indicate pairwise comparisons (Tukey’s multiple comparison test) with p values < 0.073. N=8–11/group.

Mice were examined at 4 and 5.5 months of age to determine whether sex chromosomes affect the phenotype of TREM2^R47H/+^ mice in either gonadal males or females (Figs. 2 and 3). For gonadal males at 4 months of age, chromosome sex, but not the TREM2 genotype, influenced body weight, bone mineral density (BMD, significant for total and spine BMD, and a tendency towards significance in femur BMD, *p* value 0.0703), as well as total and percent fat and lean mass (**Supplementary Table 1**). An interaction between chromosome sex and the TREM2 mutation was found for femur BMD in gonadal males (Fig. 2A). Post-hoc analyses showed that BMD was higher in XXT compared to XYT mice only for TREM2^R47H/+^ mice. Chromosome sex also influenced lean and fat mass, without post-hoc differences when 2 groups were compared, and without effects of the TREM2 variant (Fig. 2C and **Supplementary Table 1**). Post-hoc analyses showed higher body weight, and fat mass (total and percent), and lower percent lean mass for gonadal male XX mice compared to XYT only for mice expressing the wild type TREM2 gene

By 5.5 months of age, the chromosome sex effect was maintained for body weight and composition, with the only exception of a tendency towards significance for lean body mass (**Supplementary Table 1**). Further, while body weight was not different at 5.5 months of age for wild type TREM^+/+^;XYT compared to TREM^+/+^;XXT mice, it was higher for TREM^R47H/+^;XXT compared to TREM^R47H/+^;XYT mice (Fig. 3A). BMD was not different among groups, with only a tendency towards statistical difference in the chromosomal sex x TREM2 genotype for spine BMD (Fig. 3B, **Supplementary Table 1**). On the other hand, total and percent fat mass were higher, and percent lean body mass was lower for XXT compared to XYT mice, independently of the TREM2 genotype (Fig. 3C).

When the percent change between 4 and 5.5 months of age was calculated, most differences were lost for gonadal males, with only a tendency towards chromosome sex effect in body weight, and significantly lower percent lean mass in XXT mice compared to XYT (**Supplementary Fig. 1A-C** and **Supplementary Table 2**). There was a significant interaction between chromosome sex and the TREM2 genotype for femur BMD, but no differences following the post-hoc analyses.

Less differences were found for gonadal females, with BMD, but not other measurements, influenced by chromosome sex at 4 months of age (Fig. 2D-F, **Supplementary Table 1**). While no differences were detected in body weight and composition (Fig. 2D and 2F), total and spine BMD were overall lower in XYO mice compared to XXO, whereas femur BMD was only lower for XYO mice expressing wild type TREM2 compared to XXO mice of the same TREM2 genotype in 4-month-old gonadal females (Fig. 2E). By 5.5 months of age, no differences in body weight and composition (with only a tendency towards significance for chromosome sex in total lean mass, p = 0.07) were found (Fig. 3D-F, **Supplementary Table 1**). On the other hand, chromosome sex effects on femur BMD remained significant with lower levels for TREM^R47H/+^;XYO mice compared to XXO mice of the same TREM2 genotype (Fig. 3E, **Supplementary Table 1**).

When the percent change was calculated between 4 and 5.5 months of age for gonadal females, unlike gonadal males, body weight was significantly affected by chromosome sex, with lower values for XXO mice compared to XYO mice independently of the TREM2 genotype (Supplementary Fig. 1D and Supplementary Table 2). On the other hand, there were no chromosome sex- or TREM2 genotype-dependent differences for the percent change in bone mineral density (Supplementary Fig. 1E, Supplementary Table 2). Further, chromosome sex influenced total lean mass (lower for XXO compared to XYO, independent of the TREM2 genotype), and there was a significant chromosome sex x TREM2 genotype interaction for the percent change in total and percent fat mass and percent lean mass (but not percent change in total lean mass), with lower values for TREM2^R47H/+^;XYO compared to TREM2^+/+^;XYO, but higher for the TREM2 mutants compared to wild types in XXO animals for fat mass, and the opposite results for percent lean body mass, although none of the post-hoc differences reached significance (**Supplementary Fig. 1F**, **Supplementary Table 2**).

### Chromosome sex has different effects on trabecular bone structure in gonadal males versus female mice.

**Figure 4:**
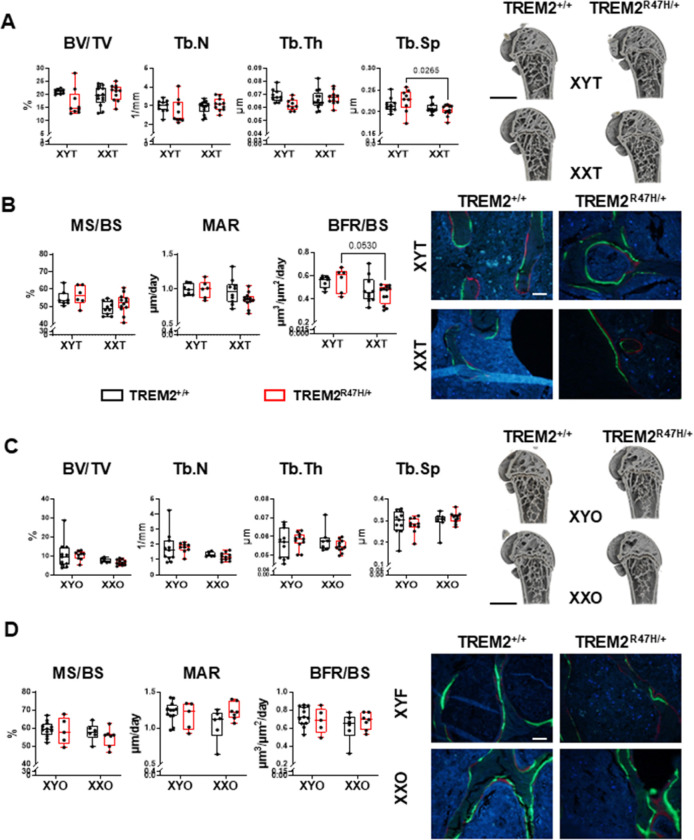
TREM2^R47H/+^ mice exhibit differences in trabecular bone dependent on the chromosome sex in gonadalmales. Trabecular micro-computed tomography (μCT) parameters, relative bone volume over tissue volume (BV/TV), trabecular number (Tb.N), trabecular thickness (Tb. Th), and trabecular spacing (Tb.Sp), (**A,C**) and bone formation parameters, mineralzing surface per bone surface (MS/BS), mineral apposition rate (MAR), and bone formation rate per bone surface (BFR/BS), (**B,D**) were measured in the distal femur of gonadal males (**A,B**) and females (**C,D**) mice at 5.5 months. Representative 3D renderings of the μCT scans (scale bar indicates 1mm) and the bone sections used to measure the histomorphometric parameters (scale bar indicates 40μm) are shown on the right. Box boundaries indicate 25^th^ to 75^th^ percentile, the horizontal lines correspond to the median, vertical lines indicate minimum to maximum, and each dot corresponds to an individual sample. p values for the two-way ANOVA analyses, with chromosomal sex and TREM2 genotype as the 2 variables, is shown in **Supplementary Table 1**. Lines indicate pairwise comparisons (Tukey’s multiple comparison test) with p values < 0.073. N=6–13/group.

We next examined the structure of the bone at the distal femur using *ex vivo* micro-computed tomography (μCT) at 5.5 months of age. For gonadal males, only trabecular separation (Tb.Sp) was affected by chromosome sex, with a significant chromosome sex x TREM2 genotype interaction for relative bone volume over tissue volume (BV/TV) and trabecular thickness (Tb.Th) (**Supplementary Table 1**). However, the only pairwise significant difference was found for Tb.Sp, with lower values for XXT compared to XYT only for TREM^R47H/+^ mice (Fig. 4A). These differences are not explained by alterations in bone formation rate parameters, since mineralizing surface (MS/BS, a measurement of the bone surface occupied by active osteoblasts) and bone formation rate (BFR/BS, a measurement of the combination of osteoblast number and activity) were overall lower, and mineral apposition rate (MAR, a measure of the activity of the osteoblasts) showed a tendency towards lower values in XXT mice compared to XYT mice (Fig. 4B and **Supplementary Table 1**). Pairwise comparisons showed only a tendency towards lower BFR/BS in XXT compared to XYT mice, only for TREM2 mutants. These pieces of evidence point toward a negative effect on the number and activity of the osteoblasts in the XXT mice that does not yet reflect into a negative effect on bone mass at the structural level in the 5.5-month-old mice. However, the serum levels of markers of bone formation P1NP or resorption CTX-1 were unchanged either when chromosome sex or TREM2 genotypes were compared in gonadal male mice (Supplementary Fig. 2, Supplementary Table 2). This suggests that the changes in osteoblasts and osteoclast activity, if any, do not reach the threshold required to detect changes in the circulation.

**Figure 5:**
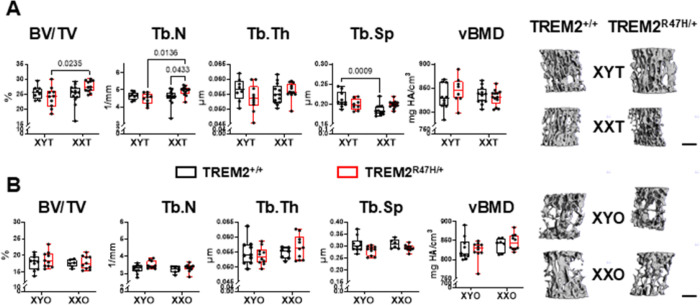
Vertebra microstructure is differentially affected by chromosome sex and TREM2 genotype, depending on gonadal sex. Trabecular micro-computed tomography (μCT) parameters, relative bone volume over tissue volume (BV/TV), trabecular number (Tb.N), trabecular thickness (Tb. Th), trabecular spacing (Tb.Sp) and volumetric bone mineral density (vBMD), were measured in the 5^th^ lumbar vertebrae of gonadal males (**A**) and females (**B**) mice at 5.5 months of age. Representative 3D renderings of the μCT scans (scale bar indicates 0.5 mm) are shown on the right. Box boundaries indicate 25^th^ to 75^th^ percentile, the horizontal lines correspond to the median, vertical lines indicate minimum to maximum, and each dot corresponds to an individual sample. p values for the two-way ANOVA analyses, with chromosomal sex and TREM2 genotype as the 2 variables, is shown in **Supplementary Table 1**. Lines indicate pairwise comparisons (Tukey’s multiple comparison test) with p values < 0.073. N=6–13/group.

Chromosome sex and its interaction with the TREM2 genotype had a higher impact on trabecular bone parameters in the lumbar vertebra in gonadal males at 5.5 months of age (Fig. 5A, **Supplementary Table 1**). These effects were evidenced by a significant chromosome sex effect on Tb.Sp, and a chromosome sex x TREM2 genotype interaction for BV/TV and trabecular number (Tb.N) and Tb.Sp. Further, TREM2^R47H/+^;XXT mice have higher BV/TV than XYT mice of the same TREM2 genotype, and higher Tb.N than both TREM2^R47H/+^;XYT and TREM2^+/+^;XXT, while only TREM2^+/+^;XXT mice have lower Tb.Sp compared to TREM2^+/+^;XYT littermates. No differences were detected either by the overall ANOVA or by the post-hoc test in trabecular thickness or volumetric bone mineral density (vBMD) in the lumbar spine.

For gonadal females, chromosome sex was the only variable that affected distal femur bone structure, with significant effects on BV/TV and Tb.N, lower for XXO compared to XYO mice, independently of the TREM2 genotype (Fig. 4C, **Supplementary Table 1**). However, no changes in bone formation parameters were detected for the gonadal female mice (Fig. 4D). Trabecular bone parameters in the lumbar vertebrae were affected by the TREM2 genotype, with overall higher Tb.N and lower Tb.Sp in TREM2^R47H/+^ compared to wild type mice and no post-hoc differences (Fig. 5B, **Supplementary Table 1**). On the other hand, we found a trend towards a significant chromosome sex effect in vBMD, higher in XXO compared to XYO mice. Further, chromosome sex influenced the levels of CTX-1 (a circulating marker that re ects osteoclast activity and bone resorption), which was lower in XXO mice compared to XYO mice, with a significant difference only for TREM2^R47H/+^ mice at 5.5 months of age, but not at 4 months (Supplementary Fig. 2 and Supplementary Table 2). On the other hand, no differences were detected on the bone formation marker, P1NP, in gonadal females at either age. Altogether, our data suggest that lower bone formation or higher bone resorption (or a combination of both) at an earlier age is responsible for the differences in bone mass in the 5.5-month-old gonadal female mice. Further, these pieces of evidence suggest the possibility that XXO mice could develop a higher bone mass phenotype compared to the XYO later in life.

### Chromosome sex has a higher influence on cortical bone parameters in gonadal females, compared to gonadal males, independently of the TREM2 genotype.

**Figure 6:**
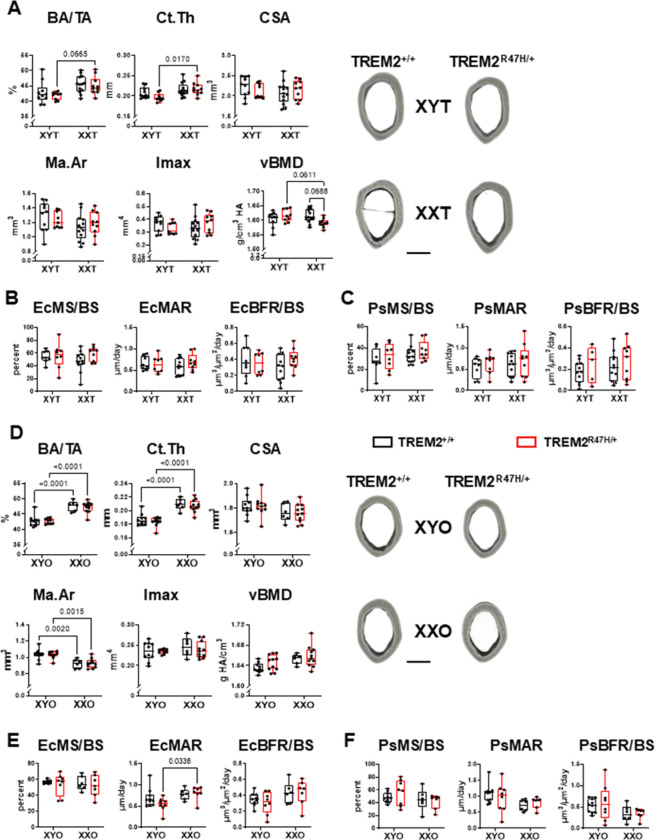
Chromosomal sex affects femoral cortical bone mass in gonadal females independently of the TREM2 genotype. Cortical micro-computed tomography (μCT) parameters, relative bone area over tissue area (BA/TA),cortical thickness (Ct.Th), cross-sectional area (CSA), marrow area (Ma.Ar), maximum moment of inertia(Imax), and volumetric bone mineral density (vBMD), (**A,D**) were measured in the femoral mid-diaphysisat 5.5 months of age. Dynamic histomorphometric parameters, mineralizing surface per bone surface(MS/BS), mineral apposition rate (MAR), and bone formation rate per bone surface (BFR/BS), weremeasured in the same site on the endocortical (Ec, **B,E**) and periosteal (Ps, **C,F**) surfaces. Data fromgonadal male (**A-C**) and female (**D-F**) mice are plotted separately. Representative 3D renderings of theμCT scans (scale bar indicates 0.5 mm) are shown on the right. Box boundaries indicate 25th to 75thpercentile, the horizontal lines correspond to the median, vertical lines indicate minimum to maximum,and each dot corresponds to an individual sample. p values for the two-way ANOVA analyses, withchromosomal sex and TREM2 genotype as the 2 variables, is shown in **Supplementary Table 1**. Linesindicate pairwise comparisons (Tukey’s multiple comparison test) with p values < 0.073. N=6–13/group.

For gonadal males, cortical bone area over tissue area (BA/TA) and cortical thickness (Ct.Th) were influenced by chromosome sex, with a tendency towards higher values for BA/TA and a significantly higher Ct.Th for XXT mice compared to XYT only for TREM2^R47H/+^ mice (Fig. 6A, **Supplementary Table 1**). On the other hand, no differences among groups were found for cross-sectional area (CSA), marrow cavity area (Ma.Ar), and maximum moment of inertia (Imax). However, there was a chromosome sex x TREM2 genotype interaction for cortical vBMD, with tendencies towards lower values for TREM2^R47H/+^;XXT mice compared to wild type XXT and to TREM2^R47H/+^;XYT. These differences were not associated with changes in BFR/BS either on the periosteal or endocortical surfaces for the gonadal males at 5.5 months of age (Fig. 6B and C and **Supplementary Table 1**).

Gonadal females experienced chromosomal sex effects for all parameters measured in the femoral mid-diaphysis, except for Imax (**Supplementary Table 1**). BA/TA and Ct.Th displayed higher values in XXO mice, whereas Ma.Ar was lower compared to their XYO counterparts for both TREM2 genotypes (Fig. 6D). CSA was lower, and vBMD was higher for XXO mice compared to XYO, without pairwise differences detected after the post-hoc tests. Higher bone formation on the endocortical bone surface and lower bone formation on the periosteal bone surface and, both associated with higher mineral apposition rate (a reflection of higher osteoblast activity), can explain the lower CSA and Ma.Ar, respectively (Fig. 6E and F). Nevertheless, the TREM2 genotype did not affect the cortical bone phenotype of gonadal female mice.

**Figure 7:**
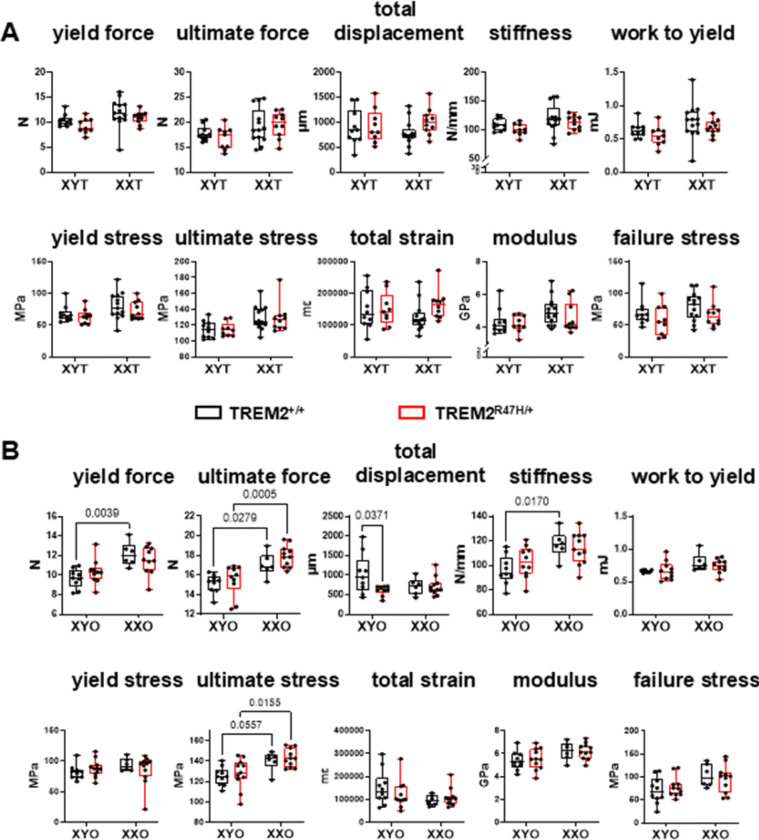
Chromosomal sex affects mechanical properties in gonadal male and female mice. Three-point bending test was performed on the femora of gonadal male (**A**) and female (**B**) mice. Parameters were either calculated directly from the strain–displacement curves or corrected by geometric measurements. Box boundaries indicate 25^th^ to 75^th^ percentile, the horizontal lines correspond to the median, vertical lines indicate minimum to maximum, and each dot corresponds to an individual sample. p values for the two-way ANOVA analyses, with chromosomal sex and TREM2 genotype as the 2 variables, is shown in **Supplementary Table 1**. Lines indicate pairwise comparisons (Tukey’s multiple comparison test) with p values < 0.073. N=6–13/group.

To assess the mechanical properties of the cortical femoral bone at the mid-diaphysis, 3-point bending tests were carried out (Fig. 7). Chromosome sex influenced most parameters measured for mice of either gonadal sex, with overall higher values for wild type and TREM2^R47H/+^;XX mice compared to XY mice, a difference most evident in gonadal female animals (Fig. 7 and **Supplementary Table 1**). While no pairwise differences were found for gonadal male mice (Fig. 7A), TREM2^+/+^;XYO exhibited lower yield and ultimate force, stiffness, and ultimate stress compared to TREM2^+/+^;XXO, whereas TREM2^R47H/+^;XXO showed higher ultimate force and ultimate stress compared to XYO of the same TREM2 genotype (Fig. 7B). In addition, the expression of the TREM2 variant led to lower total displacement compared to wild type mice only for XYO animals.

### Expression of the TREM2-R47H variant leads to higher skeletal muscle mass only in XXT mice.

In spite the lack of TREM2 expression in skeletal muscle from wild type and TREM2^R47H/+^ mice [17], we found a chromosome sex x TREM2 genotype interaction for tibialis anterior (TA) and quadriceps with a tendency towards significance for gastrocnemius muscle in gonadal males (**Supplementary Table 2**). Post-hoc analyses showed higher weight for TREM2^R47H/+^ muscles compared to TREM2^+/+^ mice for XXT mice - except for extensor digitorum longus (EDL) and soleus muscles (**Supplementary Fig. 3A**). The interaction between the 2 variables was lost when the weight of the muscles was corrected by the body weight (**Supplementary Table 2**). Instead, the corrected weight of all skeletal muscles tested, except the EDL and the soleus muscles, was lower in XXT mice compared to XYT mice, with significant post-hoc differences between TREM2^+/+^ mice for the gastrocnemius, TA, and quadriceps muscles, and between TREM2^R47H/+^ for the soleus muscle (**Supplementary Fig. 3A** and **Supplementary Table 2**). For gonadal females, we found significant interactions between gonadal sex and TREM2 genotype for the total weight of the EDL and quadriceps muscles. The interaction was only maintained for the quadriceps muscle when the mass of the muscle was corrected by body weight. Yet, no pairwise differences were found for any muscle isolated from gonadal female mice.

### Chromosomal sex and its interaction with the TREM2 genotype influence organ weight in gonadal male and female FCG mice.

Organs were collected at the time of euthanasia (5.5 months of age) and immediately weighed. Chromosome sex x TREM2 genotype interaction was significant for both the total and corrected brain weight, whereas chromosome sex affected only the corrected weight (Supplementary Table 2). The brain was lighter in TREM2^R47H/+^;XYT and TREM2^+/+^;XXT mice compared to TREM2^+/+^;XYT for the total weight and only for TREM2^+/+^;XXT mice for the corrected weight (Supplementary Fig. 4). Total kidney and spleen weight and corrected spleen weight were affected by chromosome sex in both gonadal males and females, with higher values for the kidneys and lower values for the spleens in XX mice, compared to XY animals, independently of the gonadal sex (Supplementary Fig. 4 and Supplementary Table 2). Pairwise comparisons showed differences in total spleen weight (higher in XYT wild type compared to XXT wild type mice, and in XYO TREM2 mutants compared to TREM2^R47H/+^;XXO mice. The corrected spleen weight was lower in XXT mice of either TREM2 genotype compared to the respective XYT mice, whereas the same differences as for total spleen weight were found for the corrected organ weight (Supplementary Fig. 4). While there was a trend towards significance in the corrected heart weight for gonadal males, no other variable was found to affect the organ (Supplementary Table 2). There was also a significant difference between chromosome sex and the TREM2 genotype for the corrected liver weight for gonadal female mice, without any differences detected using the post-hoc test. No differences in liver weight for gonadal males or in brain and heart weight were found for gonadal females. Overall, these pieces of data indicate that the TREM2 mutant has minor effects on the weight of the organs at 5.5 months of age.

## Discussion

Previous studies have shown that expression of the R47H variant of the TREM2 gene leads to differential effects in males compared to female mice in bone and skeletal muscle [17]. Thus, female, but not male mice, exhibit reduced bone mass accrual at 12 months of age, with low bone mass and strength compared to wild type littermates at 12 months. Further, the impact of gonadectomy on the musculoskeletal system also depends on the sex of the mice in TREM2 mutants, with minimal effects of testes removal, but an ameliorated phenotype in TREM2^R47H/+^ mice compared to wild type littermates following ovariectomy [13].

Previous studies of our laboratory showed that in addition to gonadal sex, the presence of one X and one Y versus two X chromosomes can influence the musculoskeletal system in growing, adult, and aged FCG mice [16, 22]. These studies showed for the first time that the chromosome sex complement affects bone mass and strength, as well as skeletal muscle mass, body weight, and body composition in 2-, 4-, and 20-month-old mice. However, whether the sexual dimorphic consequences of expression of the TREM2 variant depend not only on the presence of testes versus ovaries, but also on the sex chromosome complement, was heretofore unknown.

We therefore aimed to determine whether the TREM2-R47H variant altered the musculoskeletal phenotypic differences in animals sharing gonadal sex, but with different sex chromosome complements [15]. We found that whereas the presence of one X and one Y chromosomes versus two X chromosomes has a profound effect on body weight and composition and bone mass, in particular in gonadal male mice and on cortical bone structure and strength in gonadal female mice, the expression of the TREM2-R47H variant modified the differential effects of chromosome sex complement in mice of either gonadal sex.

The current study reproduced some of the differences in body weight and composition and bone mineral density as our published studies using the four core genotypes mice at 4 months of age (expressing wild type TREM2) [16]. These differences include lower body weight and total fat mass and higher percent lean body mass for XYT versus XXT TREM2^+/+^ mice, and lower femoral bone mineral density for XYO compared to XXO TREM2^+/+^ mice. Interestingly, the differences were not reproduced, whereas bone mineral density became different when the chromosome sex effects were studied within the TREM2^R47H/+^ gonadal male mice. This evidence suggests that the TREM2-R47H variant can modulate the sex chromosome-dependent musculoskeletal effects. By 5.5 months of age, differences in body weight were only found in TREM2^R47H/+^ mice, whereas mice of both TREM2 genotypes showed similar differences in body composition. However, the chromosome sex-dependent differences were mostly absent when the percent change in body weight and composition and bone mass was calculated between the 2 ages. These pieces of evidence suggest that in the presence of the TREM2-R47H variant, the chromosome sex effects are delayed compared to those expressing the wild type gene and can only be detected in older mice.

Overall, our data shows that the expression of the TREM2-R47H variant affects consequences of the presence of one X and one Y chromosome versus two X chromosomes in a manner that depends on the gonadal sex. These effects also depend on the bone studied and whether trabecular or cortical bone was analyzed. Thus, the presence of the TREM2-R47H variant has significant effects on bone structure and bone formation in the trabecular compartment of the distal femur and the lumbar vertebrae in gonadal males, whereas there was an effect dependent on the TREM2 genotype in the structure of the 5th lumbar vertebrae without any pairwise differences in gonadal female mice. For cortical bone, gonadal males and females showed differences in the structure between XY and XX mice, but the differences did not depend on the TREM2 genotype in gonadal females, where they were only seen in TREM2^R47H/+^ mice in gonadal males. Femoral mechanical properties, on the other hand, were affected by the chromosome sex and a tendency towards chromosome sex x TREM2 genotype interaction for total displacement only in gonadal females. Most of the differences observed in the wild type mice do not reproduce our previous findings in 4-month-old mice [16], suggesting the changes either were lost or appear between 4 and 5.5 months of age.

We previously showed that gonadal sex alters the musculoskeletal phenotype of mice expressing the R47H variant of TREM2, a gene that is associated with increased risk of developing AD in humans [2–4, 17]. Further, we showed that the response to gonadectomy also varies depending on the gonadal sex in TREM2^R47H/+^ mice [13]. Herein, we aimed to determine whether chromosome sex also influences the musculoskeletal phenotype of TREM2^R47H/+^ mice within each gonadal sex. We found similar responses to the TREM2 variant in XX and XY mice independently of whether they have ovaries or testes, with a few exceptions. Thus, in gonadal males, TREM2^+/+^>TREM2^R47H/+^ for Tb.N and volumetric cortical bone mineral density, and TREM2^+/+^<TREM2^R47H/+^ for TA and quadriceps muscle weights, only different in XXT mice, whereas brain weight is lower in TREM2^R47H/+^;XYT mice. In gonadal females, the only difference between TREM2^+/+^ control mice and mice expressing the TREM2 variant was found for femoral total displacement, higher in TREM2^+/+^ compared to TREM2^R47H/+^, only in XYO mice. Based on this data, we can conclude that the sex chromosome complement only minimally alters the phenotype resulting from the expression of the R47H TREM2 variant.

However, the data can also be interpreted by analyzing how the expression of the TREM2 variant alters the chromosome sex effects in gonadal males and females. Under this perspective, we can conclude that the musculoskeletal phenotypes of XX and XY are modulated in the presence of the TREM2 variant in a manner that also depends on the gonadal sex. Future studies are needed to determine the cellular and molecular basis of the interaction between chromosome sex and TREM2 genotype in the musculoskeletal system.

In summary, our study shows for the first time that the musculoskeletal differences observed in FCG mice can be modulated by expression of a gene variant known to have sex dimorphic effects in bone and skeletal muscle, in a manner that depends on the gonadal sex of the mice. Future studies are needed to determine the cellular and molecular basis for these differences.

## Supplementary Material

This is a list of supplementary files associated with this preprint. Click to download.

• RamirezetalSupplfigurelegends.docx

• SupplFig.2.tif

• SupplFig.3.tif

• SupplFig.1.tif

• SupplFig.4.tif

• Suppltable1.pdf

• Suppltable2.pdf

## Figures and Tables

**Figure 8:**
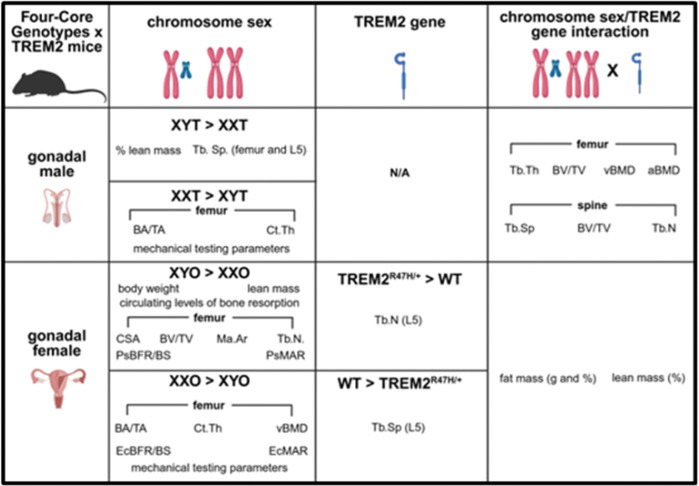
Chromosone sex differentialy influence the skeletal phenotype of TREM2^R47H/+^ mice in gonadal malesand females. The scheme depicts the findings of the current manuscript.

## Data Availability

All data generated and analyzed in this study are contained within the article and its supplementary les. The datasets analyzed during the current study are available from the corresponding author on reasonable request.

## Highlights

- The role of chromosome sex on the phenotype of mice expressing a TREM2 variant associated with AD depends on the gonadal sex
- Gonadal females exhibit more chromosomal sex effects than gonadal males for cortical bone in the mid-diaphysis.
- Gonadal males are affected by chromosomal sex and its interaction with the TREM2 variant in 5^th^ lumbar vertebrae, while only the TREM2 genotype exerts an effect in gonadal females.

## Abbreviations

TREM2: Triggering Receptor Expressed on Myeloid Cells 2
AD: Alzheimer’s disease
FCG: four core genotypes
DXA: dual X-ray absorptiometry
μCT: microcomputed tomography
R47H: histidine for arginine replacement in positioion 47
WT: wild type
P1NP: Procollagen Type 1 N-terminal propeptide
CTX-1: C-terminal telopeptide of type 1 collagen
EOAD: early-onset Alzheimer’s disease
LOAD: late-onset Alzheimer’s disease
BMD: bone mineral density
T: testes
O: ovaries
ROIs: regions of interest
BV/TV: relative bone volume
Tb.Sp: trabecular separation
Tb.Th: trabecular thickness
MS/BS: mineralizing surface
BFR/BS: bone formation rate
MAR: mineral apposition rate
Tb.N: trabecular number
vBMD: volumetric bone mineral density
BA/TA: cortical bone area over tissue area
CSA: cross-sectional area
Ma.Ar: marrow cavity area
Imax: maximum moment of inertia
Ct.Th: cortical thickness
